# Study on the Toxicological Impacts of Intraperitoneal Microcystin-LR Injection on GIFT Tilapia (*Oreochromis niloticus*) Through Multi-Omics Analysis

**DOI:** 10.3390/antiox14030296

**Published:** 2025-02-28

**Authors:** Haizheng Wu, Haojun Zhu, Quanjie Li, Jiancao Gao, Jinliang Du, Liping Cao, Yi Sun, Gangchun Xu

**Affiliations:** 1 Wuxi Fisheries College, Nanjing Agricultural University, Wuxi 214081, China; whz834591099@163.com (H.W.); dujinliang@ffrc.cn (J.D.); caoliping@ffrc.cn (L.C.); 2 Key Laboratory of Freshwater Fisheries and Germplasm Resources Utilization, Ministry of Agriculture and Rural Affairs, Freshwater Fisheries Research Center, Chinese Academy of Fishery Sciences, Wuxi 214081, China; zhuhaojun@ffrc.cn (H.Z.); liquanjie@ffrc.cn (Q.L.); gaojiancao@ffrc.cn (J.G.); sunyi@ffrc.cn (Y.S.)

**Keywords:** microcystin-LR, GIFT tilapia, oxidative stress, intestinal microbiota, SCFAs

## Abstract

This study employed multi-omics analysis to systematically evaluate the toxic effects of intraperitoneal injection of MC-LR on GIFT tilapia. The results showed that 96 h post injection, serum levels of aspartate GOT, GPT, LYZ, T-AOC, and SOD significantly decreased (*p* < 0.05). In contrast, hepatic levels of CAT and MDA significantly increased. The 16S rDNA sequencing method revealed a significant reduction in the α diversity of the intestinal microbiota. At the phylum level, the relative abundances of Firmicutes and Bacteroidota significantly decreased; at the genus level, several genera, including *Bacteroides* and *Pseudomonas*, also exhibited significant changes. Functional prediction indicated that the affected pathways were primarily related to metabolism and disease. Additionally, targeted metabolomics analysis showed a significant decrease in the levels of several SCFAs, such as butyric acid. Correlation analysis further elucidated the complex interactions between the intestinal microbiota, biochemical indicators, and SCFA metabolism. Overall, the study demonstrated that MC-LR induced oxidative stress and liver damage and led to intestinal microbiota imbalance and metabolic dysfunction in GIFT tilapia.

## 1. Introduction

The increasing eutrophication of water bodies has led to frequent occurrences of harmful algal blooms, which has become a widespread environmental issue globally [1]. After cyanobacteria die, a large amount of toxins are released, among which microcystins (MCs) are the most representative class. MCs are a class of monocyclic heptapeptide compounds with stable chemical properties and strong heat resistance. To date, more than 270 isomers have been discovered, with MC-LR exhibiting the highest toxicity [2]. Studies have shown that the concentration of MCs in surrounding water bodies significantly increases during the outbreak of harmful cyanobacterial blooms [3]. In a manmade culture pond for crucian carp *Carassius auratus* in Tianjin, China, where cyanobacterial blooms frequently occur, the total concentration of extracellular MCs in the water ranged from 1.16 to 3.66 μg L^−1^ from May to November; the concentration of MCs in suspended particles (mainly cyanobacteria) in the water was 0.64–13.98 μg g^−1^, and the concentration of MCs in the sediment was 1.34–5.90 μg g^−1^ [4]. In coastal locations in Jiangsu Province, China, the concentration of MC-LR in aquaculture ponds with diseased whiteleg shrimp *Litopenaeus vannamei* reached as high as 23.15 μg L^−1^ [5]. Both levels exceeded the limit set by the World Health Organization for MCs in drinking water (1 μg L^−1^) [6,7]. Furthermore, there are reports indicating that MCs can be transferred through the food chain, suggesting that they pose a certain toxic risk to organisms at higher trophic levels [8]. Therefore, as pollution intensifies, the harm posed by MCs to aquatic organisms and even humans will likewise rise.

It is well established that MCs can impact the liver [9], intestine [10], kidneys [11], sex glands [12,13], and heart [14]. The liver is the primary target organ of MCs in fish. The classic toxic mechanism caused by microcystins (MCs) is mainly achieved through the inhibition of protein phosphatases, especially type 1 and type 2A protein phosphatases (PP1 and PP2A), which eventually lead to the destruction of the cytoskeleton and cell death [15,16]. In addition, a large number of research results show that MCs can significantly affect the antioxidant systems of aquatic animals [10,17] and then trigger oxidative stress reactions in different types of aquatic animal organs. In the case of MC-LR, it can lead to mitochondrial damage and excessive accumulation of reactive oxygen species (ROS) [18]. If the antioxidants in the body cannot discharge excessive ROS out of the body in time, it will lead to lipid peroxidation, resulting in lipid damage [19]. It is worth noting that organisms have a set of antioxidant systems, in which the enzyme system is the key component that regulates the redox state. The enzyme defense system covers a variety of key enzymes, such as catalase (CAT), superoxide dismutase (SOD), glutathione peroxidase (GPX), and glutathione S-transferase (GST) [20]. It has been reported that when exposed to 10 μg/L MC-LR, the activity levels of ROS, GSH, SOD, CAT, and GSH—Px in the hepatocytes of *Cyprinus carpio* L. change significantly, ultimately inducing cell damage and leading to apoptosis [9]. Intraperitoneal injection of MC-LR at a dose of 100 μg/kg induces oxidative stress in the liver and gill tissues of *Brycon amazonicus*. MC-LR significantly affects the activities of antioxidant enzymes such as superoxide dismutase (SOD). In the liver tissue, the level of lipid peroxidation increases, while in the gill tissue, protein oxidation becomes the primary form of oxidative damage [21].

Gut microbiota serve as a first line of defense against pathogen invasion and environmental stress by relying on the integrity of their structure and the balance and diversity of the internal microbial community [22]. In the zebrafish (*Danio rerio*) experiment, a 20 μg/L MC-LR treatment significantly altered the structure of the zebrafish intestinal microbiome and increased the risk of pathogen invasion [23]. When *Ctenopharyngodon idella* was exposed to water containing MC-LR (35.8 μ g/L), the activity of SOD in the intestine of *Ctenopharyngodon idella* increased significantly. At the same time, pathological sections showed that the intestinal epithelial barrier was damaged, and the interaction network of flora was reshaped, which increased the relative abundance of some intestinal flora (*cetobacterium* and *Vibrio*) [24]. After *Procambarus clarkii* was exposed to 0, 10, and 40 μg/L MC-LR for 96 h, MC-LR reduced the richness and diversity of bacteria and significantly altered the microbial composition in the intestines of crayfish at both the phylum and genus levels [25]. Critically, the metabolites produced by gut microbiota have significant and complex effects on the host’s health status: beneficial metabolites positively promote host health, while harmful ones can cause adverse impacts. Short-chain fatty acids are generated through the fermentation of undigested dietary fiber and predominantly encompass acetate, propionate, and butyrate, which are of significant importance for maintaining the homeostasis of the colon. Supplementation of sodium butyrate (SB) in grass carp (*Ctenopharyngodon idella*) diet increased the contents of complement components C3 and C4 and immunoglobulin (IgM) and upregulated the expression of β-defensin-1 and hepcidin, thereby improving intestinal immune function [26]. Furthermore, it has been reported that sodium acetate can increase lipid catabolism by activating the AMPK/PPAR α pathway, thereby reducing liver lipid deposition induced by a high-fat diet [27] and reducing lipid digestibility [28]. Moreover, a report indicated that following 7-day exposure to environmental cadmium at a concentration of 5 μg/L, the intestinal microbial composition of zebrafish underwent a significant alteration. Specifically, the content of acetic acid decreased remarkably, while that of isobutyric acid increased significantly [29]. Exposure of zebrafish to florfenicol at concentrations of 0, 1, 10, and 100 μg/L for 28 days led to the disruption of the intestinal flora and the inhibition of the production of short-chain fatty acids (SCFAs) [30]. China is the largest producer of tilapia in the world, second only to the four freshwater breeding species of tilapia. Genetically improved farmed tilapia (GIFT) is a genetically modified tilapia, which has the advantages of rapid growth, high fertility, and high meat yield [31]. However, to date, there is a lack of literature reporting the specific toxic effects of MC-LR on the liver and gut microorganisms as well as their metabolites in GIFT tilapia.

This study employed multi-omics analysis to comprehensively evaluate the changes in antioxidant indicators, immune function, intestinal microbiota, and metabolic profiles in serum and liver, aiming to elucidate the toxic effects of MC-LR on GIFT tilapia. The findings will enhance our understanding of the underlying toxic mechanisms of MC-LR in fish and provide a robust scientific foundation for developing strategies to prevent and mitigate its toxicity.

## 2. Materials and Methods

### 2.1. Reagents and Drugs

Microcystin-LR (purity of >95%) was purchased from the Qingdao Puruibang Bioengineering Co., Ltd., Qingdao, China. Kits for measuring alanine aminotransferase (GPT), alkaline phosphatase (AKP), aspartate aminotransferase (GOT), catalase (CAT), malondialdehyde (MDA), superoxide dismutase (SOD), total cholesterol (TC), total protein (TP), total antioxidant capacity (T-AOC), and triglycerides (TG) were sourced from the Nanjing Jiancheng Bioengineering Institute (Nanjing, China). Kits for detection of fish complement C3 (C3) and lysozyme (LYZ) were purchased from the Shanghai Yuanju Bio-Tech Co., Ltd. (Shanghai, China). (Fish antibodies included fish complement protein antibodies pre-coated on microplate plates and HRP-labeled anti-fish complement protein antibodies.) For the target metabolites, acetic acid, isobutyric acid, and butyric acid were purchased from CATO Research Chemicals Inc. (Guangzhou, China); propionic acid was obtained from AccuStandard, Inc. (New Haven, USA); valeric acid and isovaleric acid were sourced from BePure (Beijing, China); and 3-Nitrophenylhydrazine (3-NPH) (97% purity) and N-(3-dimethylaminopropyl)-N′-ethylcarbodiimide hydrochloride (EDC) (97% purity) were purchased from McLean Biotechnology Ltd. (North Yorkshire, UK).

### 2.2. Experimental Fish and Rearing Conditions

All animal experiments were performed in compliance with the guidelines set forth in the Guide for the Care and Use of Laboratory Animals [32]. A total of 160 two-month-old GIFT tilapia (66.79 ± 6.44 g, 124.25 ± 9.49 mm) from the Freshwater Fisheries Research Center of the Chinese Academy of Fishery Sciences were allowed to acclimatize in a recirculating aquaculture system for 2 weeks before the experiment commenced. During this period, the fish were fed a commercial feed (Shanghai Tianen Biotechnology Co., Ltd., Shanghai, China) twice daily, at 08:00 and 17:00. For the duration of the experiment, the water was maintained under the following conditions: dissolved oxygen concentration of >6 mg L^−1^, pH range of 7.2–7.8, NO_2_^−1^ level of <0.02 mg L^−1^, and NH₃ level of <0.05 mg L^−1^.

### 2.3. Experimental Design

The total of 160 healthy tilapia were randomly divided into two groups: a blank control group (C) and a treatment group (T), with 80 fish in each group and four replicates of 20 fish each per group. Our previous research determined the acute toxicity of MC-LR (96-h LD50) for GIFT larvae as 472.93 μg kg^−1^. During the 96 h toxicity test, a 40% dose of LD50 (about 200 μg kg^-^¹) was administered. MC-LR was first diluted with 0.86% normal saline. According to the body weight of the GIFT tilapia, intraperitoneal injection (0.05 mL/10 g body weight) was performed, and the blank control group was given 0.86% normal saline of the same volume. Mortality was recorded every 24 h. Blood, liver, and intestinal samples were collected at 12 h, 24 h, 48 h, and 96 h post experiment initiation. GIFT tilapia were anesthetized with eugenol (20 mg L^−1^), and blood samples were obtained via tail vein puncture. Blood was allowed to clot at room temperature for 30 min before centrifugation at 4 °C for 10 min at 4000 rpm to collect serum. Fish were dissected on ice, and livers were promptly collected. The collected livers were immediately immersed in liquid nitrogen for rapid freezing and subsequently stored at −80 °C for further biochemical analysis. Intestinal samples were rinsed with 0.86% saline solution, excess fat was carefully removed using forceps, and the samples were stored at −80 °C for future analysis of the intestinal microbiome and metabolomics.

### 2.4. Biochemical Indicators Testing

An appropriate amount of liver tissue was collected and mixed with 9 times its volume of normal saline (*w*/*v*). The mixture was thoroughly homogenized. Subsequently, it was centrifuged at 1800 rpm for 15 min at 4 °C, and the supernatant was collected as the liver extract. Biochemical detection kits were used to measure the activities or levels of GOT, GPT, TG, TC, AKP, T—AOC, CAT, SOD, and MDA in the liver or serum of GIFT tilapia. The levels of lysozyme (LYZ) and complement C3 in the serum were detected using ELISA kits with the double-antibody one-step sandwich method. All procedures were performed in accordance with the manufacturers’ instructions.

### 2.5. Microbiome Analysis

#### 2.5.1. Extraction of Gut Microbiota DNA and PCR Amplification

Total DNA was extracted using the E.Z.N.A. Stool DNA Kit (Omega, Mountain Lakes, NJ, USA). The V3–V4 variable region of bacterial DNA was amplified using the forward primer 341F (5′-CCTACGGGNGGCWGCAG-3′) and the reverse primer 805R (5′-GACTACHVGGGTATCTAATCC-3′). The amplified products were purified using AMPure XT beads (Beckman Coulter Genomics, Danvers, MA, USA) and quantified using Qubit (Invitrogen, Carlsbad, CA, USA). The amplified products were detected by agarose gel electrophoresis and recovered using the AMPure XT beads recovery kit. Then, the concentration was adjusted for sequencing. Paired-end sequencing with a read length of 2 × 250 bp was carried out on the Illumina NovaSeq 6000 platform (Illumina, Inc., San Diego, CA, USA; Hangzhou LC—Bio Technologies Co., Ltd., Hangzhou, China).

#### 2.5.2. Sequencing Data Analysis

By leveraging unique barcode technology, sequencing data were accurately assigned to individual samples, and sequencing adapters and primer sequences were removed. Read pair assembly was conducted using FLASH software (v1.2.8), quality control filtering was performed with fqtrim software (v0.94), and VSEARCH (v2.3.4) was utilized to detect and eliminate chimeric sequences. Subsequently, the DADA2 algorithm was employed to generate feature tables and feature sequences.

### 2.6. Targeted Metabolomics Analysis

Targeted metabolomics analysis was conducted using standard substances as references. Following the thawing of intestinal samples, each sample was precisely weighed to ensure that its mass was within 50 ± 2 mg. An appropriate volume of 80% methanol aqueous solution was then added to each sample for extraction. The mixture was subsequently centrifuged at 4 °C with a relative centrifugal force (rcf) of 20,000 g for 15 min. After centrifugation, 20 μL of the supernatant was transferred to a 1.5 mL centrifuge tube and derivatized using EDC solution and 3-NPH. Next, the initial mobile phase was added to bring the total volume to 500 μL, followed by thorough vortex mixing. Subsequently, 200 μL of the mixture were transferred to a sample vial for quantitative analysis via liquid chromatography-tandem mass spectrometry (LC-MS/MS). The experimental instruments included an AB SCIEX 4500MD triple quadrupole mass spectrometer and an AB Sciex Jasper^TM^ ultra performance liquid chromatograph (Triple/Jasper QuadTM 4500MD, AB Sciex, Shanghai, China). During the analysis, an Agilent Poroshell 120 EC-C18 2.7 μm column (2.1 × 100 mm) was employed, with the column temperature set at 40 °C and an injection volume of 2 μL. Water (A) and methanol + acetonitrile (1:1) (B) were used as mobile phases for liquid chromatography. Elution gradient settings are shown in Table S1. If the sample concentration exceeded the range of the standard curve, the sample was appropriately diluted and reanalyzed. Data from the diluted samples were used for final quantitative analysis.

### 2.7. Statistical Analysis

An independent samples *t*-test was employed to compare the means between the two groups. Results are presented as means ± standard deviation (SD). *p* < 0.05 was considered statistically significant.

α and β diversity analyses were conducted using the QIIME2 platform, and species annotation was achieved by integrating the SILVA and NT-16S databases. The LEfSe method was applied to identify significant differential species across samples, and the STAMP bioinformatics tool was used for the analysis and visualization of functional metabolic pathways. All charts and figures were generated using relevant packages in R (v3.4.4) and GraphPad Prism 8 software.

## 3. Results

### 3.1. Analysis of Biochemical Indicators

#### 3.1.1. Serum Biochemical Indicators

**Figure 1:**
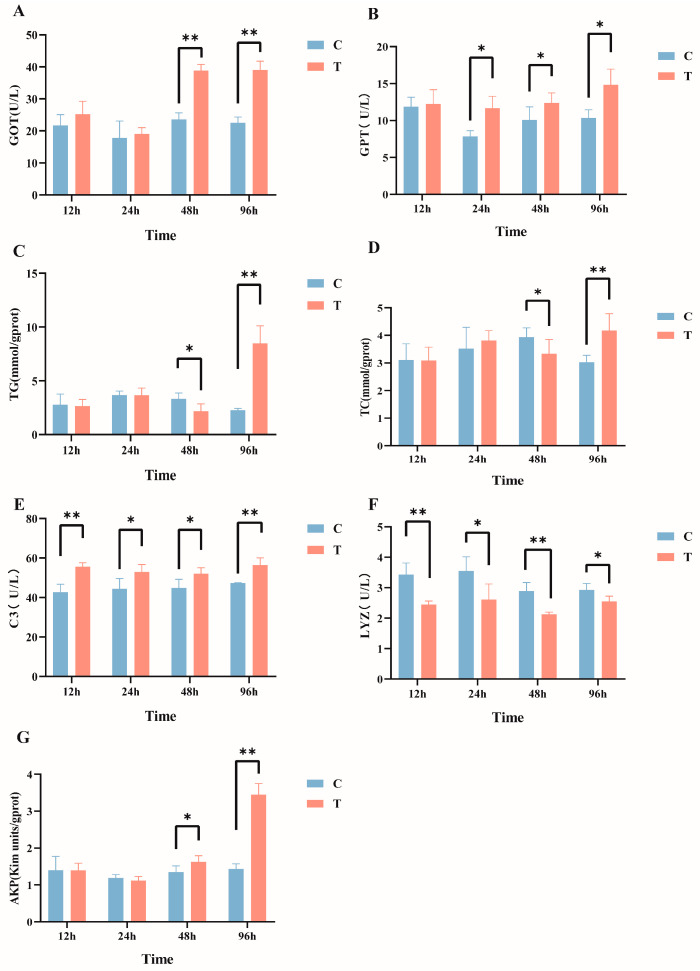
Effects of intraperitoneal injection of microcystin-LR on serum biochemical indicators in GIFT tilapia: (**A**) GOT. (**B**) GPT. (**C**) TG. (**D**) TC. (**E**) C3. (**F**) LYZ. (**G**) AKP. The results are expressed as the means ± SD (*n* = 8). * *p* < 0.05; ** *p* < 0.01. Compared with the control group.

After intraperitoneal injection of MC-LR in GIFT tilapia, multiple serum biochemical indicators exhibited significant changes. Specifically, compared with the control group, the serum GOT activity (Figure 1A) in the MC-LR treatment group was significantly increased at 48 h and 96 h post injection. The GPT levels (Figure 1B) significantly increased from 24 h to 96 h post injection. The TG (Figure 1C) and TC (Figure 1D) levels significantly decreased at 48 h and then significantly increased at 96 h post injection. Additionally, C3 levels were significantly elevated at all time points, Lyz activity (Figure 1F) was significantly reduced at all time points, and AKP activity (Figure 1G) significantly increased at both 48 h and 96 h post injection (*p* < 0.05).

#### 3.1.2. Liver Biochemical Indicators

**Figure 2:**
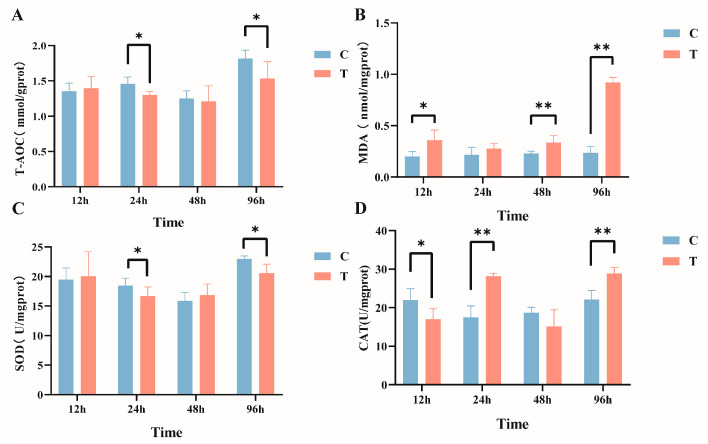
Effects of intraperitoneal injection of microcystin-LR on liver biochemical indicators in GIFT tilapia (**A**) T-AOC. (**B**) MDA. (**C**) SOD. (**D**) CAT. The results are expressed as the means ± SD (*n* = 8). * *p* < 0.05; ** *p* < 0.01. Compared with the control group.

The effects of intraperitoneal injection of MC-LR on liver biochemical indicators in GIFT tilapia are shown in Figure 2. In comparison with the control group, the activity of T-AOC (Figure 2A) manifested a significant decline within the timeframes of 24 h and 96 h, and MDA content (Figure 2B) exhibited significant increases at 12 h, 48 h, and 96 h post exposure. In addition, the activity of SOD (Figure 2C) was significantly reduced after 24 h and 96 h of treatment. The activity of CAT (Figure 2D) significantly declined at 12 h and subsequently exhibited a significant increase at 24 h and 96 h (*p* < 0.05).

### 3.2. Intestinal Microbiota

#### 3.2.1. Sequencing Depth

The sequencing results indicated that the number of raw tags in each sample ranged from 80,161 to 87,919, while the number of valid tags ranged from 71,702 to 80,547. The ASV counts per sample varied from 124 to 366, encompassing 29 phyla, 59 classes, 136 orders, 212 families, and 394 genera. The control group possessed the highest number of ASVs, totaling 1043, with 405 ASVs shared between the two groups (Figure S1A). The rarefaction curves (Figure S1B) demonstrated that as sequencing depth increased, the observed ASV counts progressively stabilized, indicating adequate and saturated sequencing coverage, thus providing a robust foundation for further analysis.

#### 3.2.2. Analysis of Taxonomic Composition of Intestinal Microbiota

Alpha diversity, which reflects species richness and evenness within the community, was quantified using indices such as Chao1, Shannon, Simpson, and Pielou. Analysis of the intestinal microbiota revealed that the Shannon, Simpson, Chao1, and Pielou indices in the treatment group were significantly lower than those in the control group (*p* < 0.05), indicating a significant reduction in microbial diversity due to MC-LR treatment (Figure S2A–D). To further investigate the effects of MC-LR-induced stress on the intestinal microbiota, we performed β-diversity analysis via principal coordinate analysis (PCoA). The results (Figure S2E) demonstrated significant differences in microbial composition between the control and treatment groups, suggesting that MC-LR exposure markedly altered the structure of the intestinal microbiota in GIFT tilapia.

**Figure 3:**
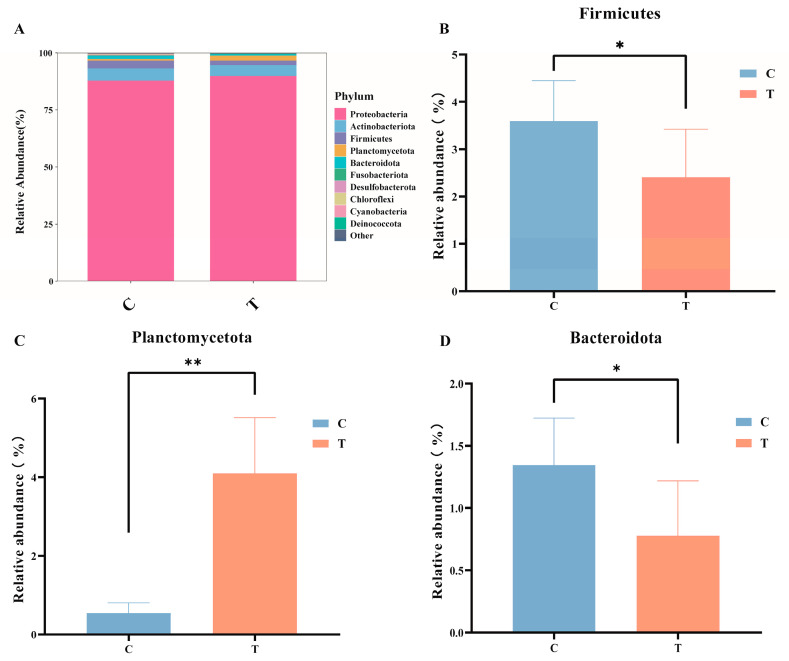
(**A**) Stacked bar chart of gut microbiota. (**B**) The variation in the relative abundance of Firmicutes. (**C**) The variation in the relative abundance of Planctomycetota. (**D**) The variation in the relative abundance of Bacteroidota. * *p* < 0.05; ** *p* < 0.01. Compared with the control group.

In the control group, the top four predominant phyla were Proteobacteria (88.02%), Actinobacteriota (5.33%), Firmicutes (3.41%), and Bacteroidota (1.34%). In contrast, in the MC-LR treatment group, the top four predominant phyla were Proteobacteria (90.02%), Actinobacteriota (4.79%), Planctomycetota (2.15%), and Firmicutes (1.93%). Compared with the control group, the relative abundance of Planctomycetota (Figure 3C) in MC-LR treatment group significantly increased, whereas the relative abundances of Firmicutes (Figure 3B) and Bacteroidota (Figure 3D) markedly decreased. It is important to highlight that Chlorofexi (0.29% and 0.02%, respectively) and Cyanobacteria (0.12% and 0.05%, respectively) were detected in both the control and treatment groups.

**Figure 4:**
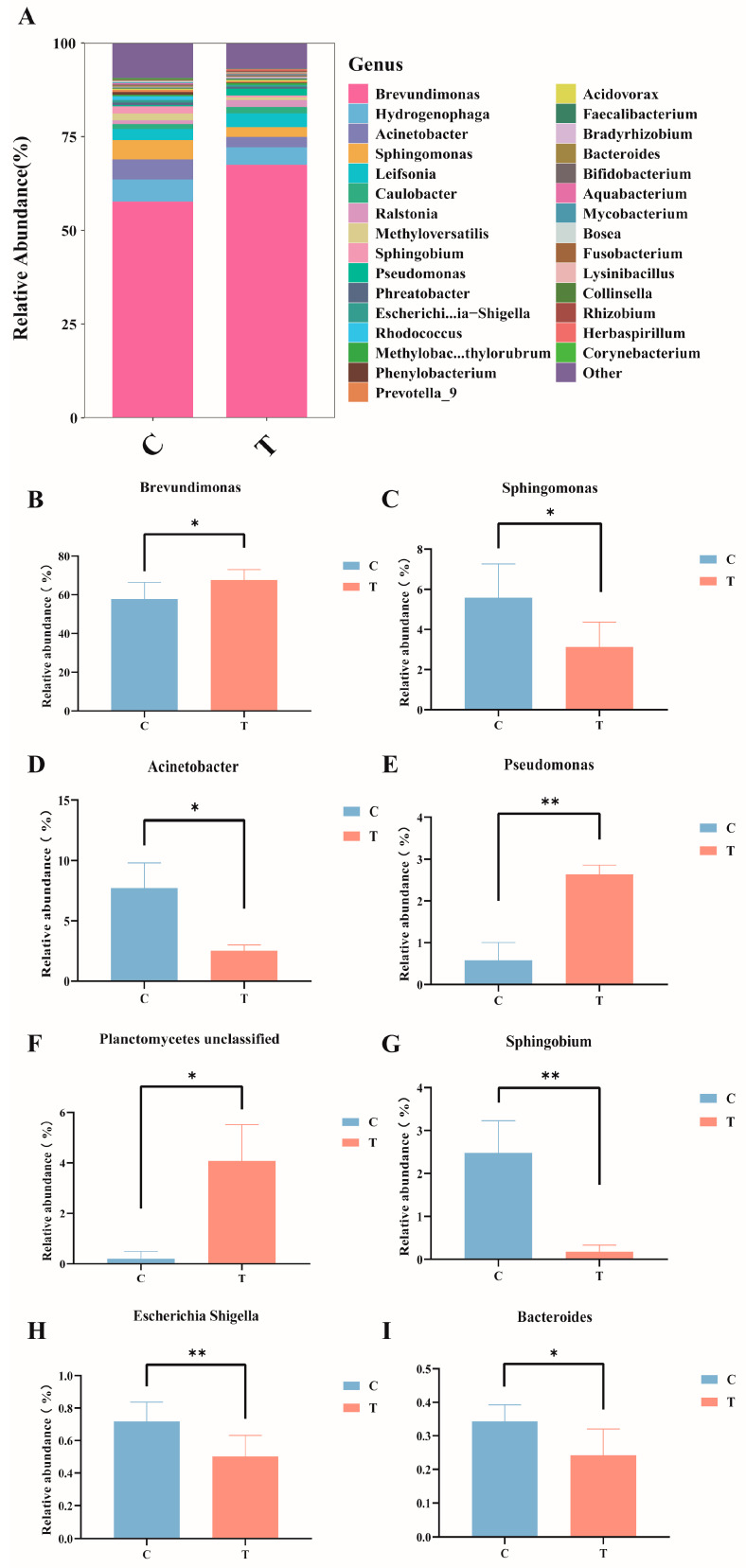
Composition and abundance of the intestinal microbiota in GIFT tilapia at the genus level. (**A**) Columnar stacked chart; (**B**) Changes in the relative abundance of *Brevundimonas;* (**C**) Changes in the relative abundance of *Sphingomonas;* (**D**) Changes in the relative abundance of *Acinetobacter;* (**E**) Changes in the relative abundance of *Pseudomonas;* (**F**) Changes in the relative abundance of *Planetomyeetes unclassified;* (**G**) Changes in the relative abundance of *Sphingobium;* (**H**) Changes in the relative abundance of *Escherichia Shigella;* (**I**) Changes in the relative abundance of *Bacteroides*. * *p* < 0.05; ** *p* < 0.01. Compared with the control group.

At the genus level (Figure 4A), in the control group, the dominant bacteria genera were *Brevundimonas* (57.81%), *Hydrogenophaga* (5.90%), *Acinetobacter* (5.37%), and *Sphingomonas* (5.12%); in the MC-LR treatment group, the dominant bacteria genera were *Brevundimonas* (67.65%), *Hydrogenophaga* (4.70%), *Leifsonia* (2.98%), and *Acinetobacter* (2.72%). Compared with the control group, the relative abundance of *Brevundimonas*, *Pseudomonas*, and *Planctomycetes_unclassified* significantly increased (Figure 4B,E,F), whereas the relative abundance of *Sphingomonas*, *Acinetobacter*, *Sphingobium*, *Escherichia-Shigella*, and *Bacteroides* significantly decreased (Figure 4C,D,G–I; *p* < 0.05).

**Figure 5:**
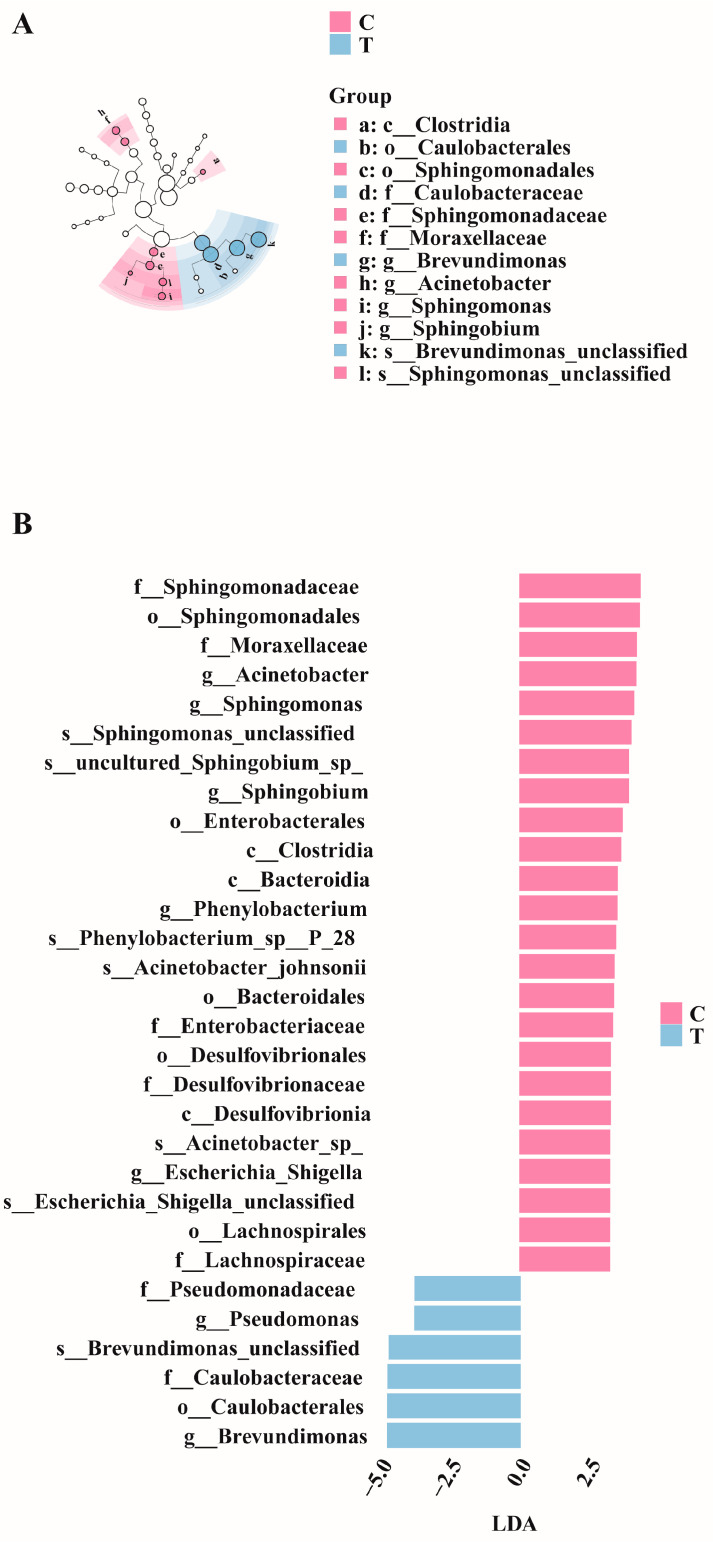
LefSe differential analysis diagrams. (**A**) Evolutionary cladogram. (**B**) Distribution bar chart (LDA > 2.5).

LEfSe analysis was conducted on the control and treatment groups. Figure 5A illustrates the significantly differentiated species across different taxonomic levels. Linear discriminant analysis (LDA) revealed that species with an LDA > 2.5 were significantly differentiated (Figure 5B). Specifically, the relative abundances of *Acinetobacter*, *Sphingomonas*, and *Phenylobacterium* were higher in the control group, whereas those of *Pseudomonas* and *Brevundimonas* were higher in the treatment group.

The PICRUSt2 software (v2.5.2) was employed for the functional prediction analysis of the gut microbiota. The KEGG level 2 pathways (Figure S3) indicated that the secondary pathways were mainly related to transcription, signal molecules and interactions, glycan biosynthesis and metabolism, transport and catabolism, and genetic information processing. Further analysis of the KEGG level 3 pathways (Figure S4) revealed that the tertiary pathways were predominantly associated with biotin metabolism, fatty acid biosynthesis, lipopolysaccharide biosynthesis proteins, linoleic acid metabolism, energy metabolism, and nucleotide metabolism.

### 3.3. The Impact of MC-LR on SCFAs in the Intestine

**Figure 6:**
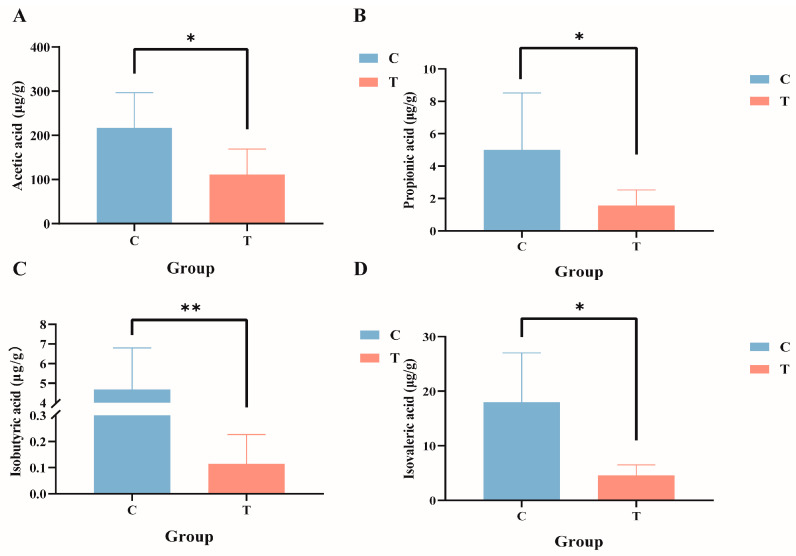
Composition of short-chain fatty acids in the gut contents of GIFT tilapia. (**A**) Acetic acid; (**B**) propionic acid; (**C**) isobutyric acid; and (**D**) isovaleric acid. * *p* < 0.05; ** *p* < 0.01. Compared with the control group.

Targeted metabolomics analysis of the intestinal tract of GIFT tilapia revealed significant reductions in the levels of acetic acid, propionic acid, isobutyric acid, and isovaleric acid (Figure 6A–D; *p* < 0.05), whereas no significant differences were observed for butyric acid, valeric acid, or caproic acid.

### 3.4. Joint Analysis

**Figure 7:**
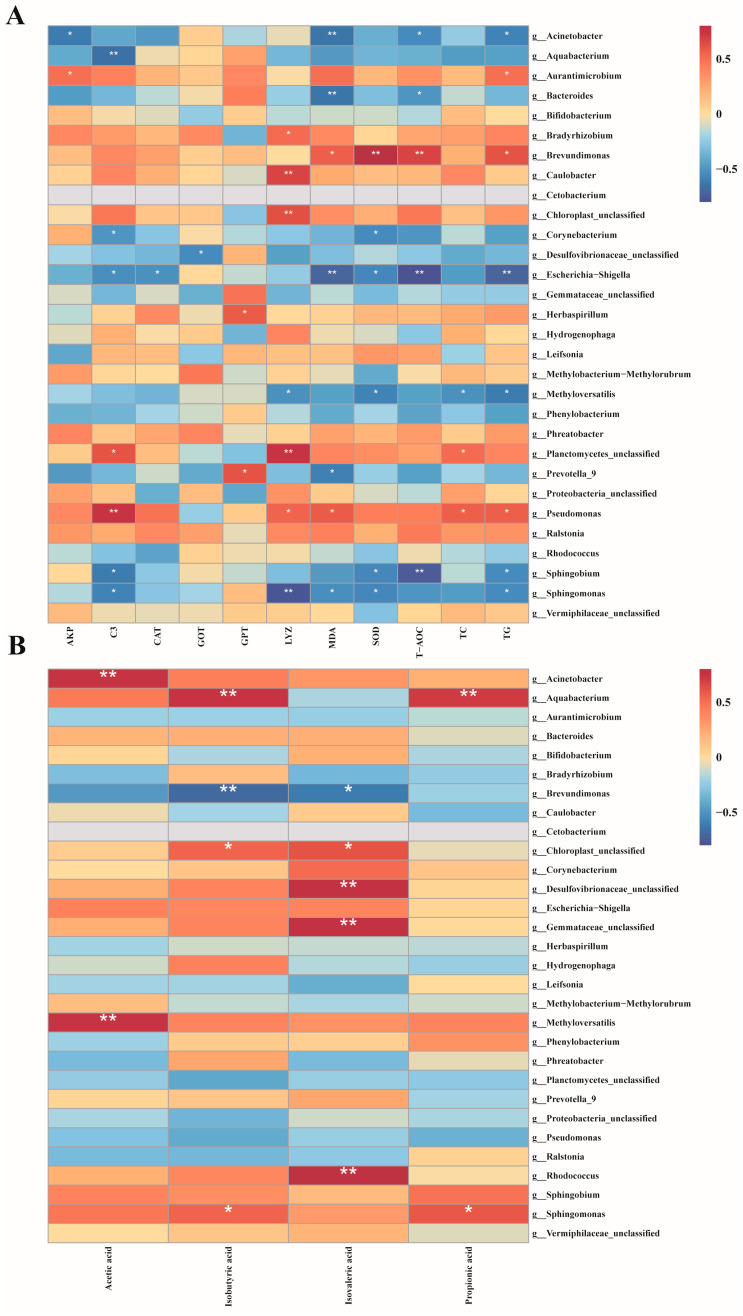
Pearson rank correlation matrix between the intestinal microbiota of GIFT tilapia after treatment and biochemical indicators, as well as SCFAs. (**A**) Correlation analysis of gut microbiota and biochemical indicators. (**B**) Correlation analysis of gut microbiota and short-chain fatty acids, * *p* < 0.05; ** *p* < 0.01.

Pearson rank correlation analysis was conducted to investigate the impact of MC-LR exposure on the relationships between biochemical indicators, SCFAs, and specific intestinal microbiota in GIFT tilapia (Figure 7). The results indicated that *Acinetobacter*, *Aquabacterium*, *Bacteroides*, *Corynebacterium*, *Escherichia-Shigella*, *Methyloversatilis*, *Prevotella 9*, *Sphingobium*, and *Sphingomonas* exhibited significant negative correlations with multiple biochemical indicators (TG, TC, GPT, MDA, LYZ, etc). In contrast, *Aurantimicrobium*, *Bradyrhizobium*, *Brevundimonas*, *Herbaspirillum*, and *Pseudomonas* demonstrated significant positive correlations with key biochemical markers such as SOD, MDA, C3, and T-AOC. Furthermore, *Acinetobacter*, *Aquabacterium*, *Methyloversatilis*, and *Rhodococcus* showed significant positive correlations with several short-chain fatty acids (SCFAs), including acetic acid and isobutyric acid. Conversely, *Brevundimonas* exhibited a significant negative correlation with isobutyric acid and isovaleric acid.

## 4. Discussion

The high frequency of outbreaks of cyanobacterial blooms caused by eutrophication presents a significant threat to the health of ecosystems and the survival of aquatic organisms [33]. Intraperitoneal injection and water-borne exposure are two common methods for studying the toxic effects of toxic substances on aquatic animals [34]. Exposure to toxins in water can maximize the simulation of the actual exposure of fish to toxic substances in the natural environment. However, water exposure has certain drawbacks. Water-borne exposure is easily interfered with by various environmental factors [35]. In contrast, intraperitoneal injection has fewer interference factors and is convenient for the study of dose-response relationship of toxicants, which is not only suitable for aquatic animal toxicology studies but also widely used in mammalian toxicology studies [36]. Therefore, this study conducted an acute attack experiment lasting 96 h by intraperitoneal injection of MC-LR, and the toxicity of MC-LR to GIFT tilapia was deeply investigated by multi-omics analysis.

### 4.1. Effect of MC-LR on Serum Biochemical Indexes

The activities of GOT and GPT in the blood are crucial indicators for assessing liver health [37]. Normally, the concentrations of these two enzymes in the blood remain at low levels; however, when exposed to hepatotoxic substances, the concentrations of these enzymes in the blood can increase significantly, serving as an important basis for judging liver damage [38]. Under laboratory conditions, the levels of GOT and GPT in common carp (*Cyprinus carpio* L.) serum increased significantly after 28 days of feeding cyanobacteria bloom at a dose of 50 μg/kg [39]. In the research on *Eriocheir sinensis*, after intravenous injection of 0.03 μg/g MC-LR, it was observed that the levels of GOT and GPT significantly increased within 12 h to 96 h [40], which is consistent with the results of this study, indicating that intraperitoneal injection of MC-LR induces liver damage in GIFT tilapia. Functional TG and TC are crucial biomarkers for assessing lipid metabolism status. Their levels directly reflect the accumulation of lipids (i.e., fats) in the body [41]. For example, when *Pelophylax nigromaculatus* was exposed to environmentally relevant concentrations of microcystin-LR (MC-LR) (0, 1, and 10 μg/L) for 21 days, the levels of triglyceride (TG) and total cholesterol (TC) increased in the liver, while they decreased significantly in the serum. Moreover, the level of total bile acid (TBA) in the serum increased significantly [42]. Chronic exposure to MC-LR also resulted in a significant increase in hepatic TG and TC in zebrafish Danio rerio, indicating a certain level of dose-dependency [43]. The results of this study are consistent with previous findings. In this study, compared with the control group, intraperitoneal injection of MC-LR for 96 h led to a significant increase in serum TG and TC levels, which suggested that MC-LR may influence lipid metabolism in tilapia, although the specific mechanisms require further investigation.

In the innate immune system of fish, the activation of immune cells relies on a variety of important molecules, among which complement enzymes and lysozyme play crucial roles. These molecules are extensively involved in antibacterial defense and immune regulation within the host, performing indispensable functions [44,45]. Specifically, complement C3, as a central component of the complement cascade, not only exhibits the highest abundance but also plays a decisive role in initiating and regulating the comprehensive activation of the complement system. Previous studies have shown that the levels of serum complement C3 became significantly elevated in two MC-LR-exposure groups of silver carp [46]. Other research found that both low and high concentrations of MC-LR led to significant elevation in serum complement C3 in male zebrafish [47]. LYZ, a key molecule in the non-specific immune system of fish, is essential for resisting the invasion of foreign pathogens. Interestingly, in this study, the lysozyme activity significantly decreased between 12 h and 96 h, indicating an immunosuppressive effect. Exposure to environmental concentrations of MC-LR (0.5 and 5 μ g/L) for 3 weeks resulted in a significant decrease in Lyz activity in *Macrobrachium rosenbergii* hepatopancreas [48], which was similar to this study. These findings indicate that MC-LR can induce innate immune imbalance in GIFT tilapia, which makes the immune defense of the body unable to function normally. As a crucial immune and metabolism-related enzyme, AKP plays an important role in the degree of an organism’s response to external environmental stress or damage. It has been reported that AKP activity in the hepatopancreas of whiteleg shrimp significantly increased under MC-LR exposure [49], and similar results were found in a study of red claw crayfish *Cherax quadricarinatus* [50]. This research revealed that AKP activity significantly increased in GIFT tilapia from 48 h to 96 h post treatment. The increase in AKP activity may be attributed to the stress response elicited by MC-LR stimulation, resulting in enhanced lysosomal membrane permeability [49].

### 4.2. Effect of MC-LR on Liver Biochemical Indexes

T-AOC is an important indicator for measuring the comprehensive efficacy of a system or substance in resisting oxidative stress, scavenging free radicals, or inhibiting oxidative reactions. Previous studies found that exposure to 100 μg L^−1^ of MC-LR in water enhanced antioxidant capacity in whiteleg shrimp [51]. Contrary to the aforementioned findings, in this study, the T-AOC in the liver of GIFT tilapia significantly decreased at 24 h and 96 h post intraperitoneal injection of MC-LR. These results suggest that MC-LR may induce oxidative stress by inhibiting the activity of specific antioxidant enzymes. Increased oxidative stress is one of the toxic effects of environmental contaminants in fish [52]. MC-LR exhibits a harmful biological effect by triggering excessive production of reactive oxygen species (ROS) in the organism, which causes cellular and tissue damage. Malondialdehyde (MDA), the end product of lipid peroxidation by ROS, can reflect the degree of oxidative damage in the organism [53]. It was found that intraperitoneal injection of 50 or 120 μg kg^−1^ of MC-LR significantly increased the MDA level in the liver of common carp [54]. In our study, MC-LR exposure led to significant elevations in MDA levels in the liver of GIFT tilapia at 12 h, 48 h, and 96 h, indicating that MC-LR induced lipid peroxidation and could result in liver damage. To counteract cellular and tissue damage caused by oxidative stress, a series of defense mechanisms are initiated in the organism, a crucial aspect of which is the activation of the antioxidant enzyme system. SOD and CAT form a tightly coordinated chain in the antioxidant process: SOD is primarily responsible for converting superoxide anions into hydrogen peroxide, while CAT subsequently decomposes hydrogen peroxide into harmless water and oxygen, jointly ensuring the effective removal of ROS within cells and thereby protecting cells from oxidative stress damage [55]. Research has found that under laboratory conditions, acute exposure to microcystin-containing toxic cyanobacteria cells significantly decreased SOD activity in the liver and kidneys of GIFT Tilapia at 24 h and 72 h; in addition, after 21 days of exposure, CAT activity in the liver and kidneys increased significantly by 2.5-fold [17]. This result is consistent with the findings of this study, indicating that MC-LR induces oxidative stress and activates antioxidant enzymes as a defense mechanism, thereby demonstrating an adaptive response to MC-LR. However, this adaptive response is insufficient to halt the progression of oxidative damage.

### 4.3. Effect of MC-LR on Intestinal Microorganisms

The gut microbiota have a pivotal role in maintaining host homeostasis and overall health. The homeostasis of intestinal function is closely associated with the dynamic balance of the intestinal microbiota. In this study, Proteobacteria, Actinobacteriota, Planctomycetota, and Firmicutes were identified as the dominant phyla 96 h after intraperitoneal injection of MC-LR. Proteobacteria are primarily responsible for regulating amino acid metabolism. Additionally, Proteobacteria include various potential pathogens, such as *Vibrio* [56], *Aeromonas hydrophila* [57], and *Edwardsiella* [58]. Actinobacteriota is usually not the most dominant in the gut microbial community, but it is often one of the four major phyla and plays an indispensable role in aquaculture [59]. Firmicutes play a crucial role in the digestion and fermentation of dietary fiber and contribute to short-chain fatty acid production. Studies have shown that exposure to MC-LR in aquatic environments significantly decreases the relative abundance of Firmicutes in the intestines of grass carp [24], and members of Bacteroidota produce enzymes to convert recalcitrant carbohydrates into small molecules (such as SCFAs) or participate in the degradation of environmental proteins [60]. It was reported that the relative abundance of Bacteroidota in the intestinal tissue of carp exposed to MC-LR was significantly decreased [61]. Members of Planctomycetota participate in the cycling of elements such as carbon, nitrogen, and sulfur. The findings of this study align with previous research. Notably, compared to the control group, MC-LR treatment resulted in a significant reduction in the relative abundance of Bacteroidota and Firmicutes, as well as a significant decrease in Planctomycetota. Fan et al. found that Chloroflexi and Cyanobacteria were detected in tilapia aquaculture pond water bodies, sediments, and tilapia intestinal tracts; cyanobacteria were shared between water and intestinal libraries, and Chloroflexi was shared between intestinal and sediment [62]. In this study, Chloroflexi and Cyanobacteria were detected in the intestines of the fish, and their relative abundance was low. We speculated that Chloroflexi and Cyanobacteria might be the result of GIFT bait or interaction with the external environment.

At the genus level, compared with the control group, the relative abundance of *Brevundimonas* and *Pseudomonas* in the intestinal samples of the MC-LR treatment group was significantly higher, whereas that of *Sphingomonas*, *Acinetobacter*, *Sphingobium*, and *Bacteroides* was markedly lower. *Brevundimonas* is a genus of Gram-negative bacteria, and some are considered opportunistic pathogens in humans, especially immunocompromised individuals, and are associated with bacteremia [63]. Certain *Pseudomonas* species, such as *P. aeruginosa*, are opportunistic pathogens that can cause diseases in a wide range of host organisms. Both *Sphingobium* and *Sphingomonas* are classified within the *Proteobacteria* and specifically fall under the class α-Proteobacteria. *Sphingobium* is a genus of Gram-negative bacteria with diverse metabolic pathways, capable of degrading various organic pollutants. The *Sphingomonas* is considered a potentially beneficial bacterium in aquaculture, capable of degrading ammonia nitrogen and nitrite [64] and inhibiting pathogens such as *Vibrio* [65]. Furthermore, several studies have demonstrated that this species exhibits considerable potential in degrading MCs [66]. Some members of the *Acinetobacter* are opportunistic pathogens that can cause infections when aquatic conditions deteriorate or host immunity is compromised. However, research has demonstrated that certain strains within this genus possess the capability to degrade both Microcystis and the microcystins it produces [67]. *Bacteroides* promotes the repair of intestinal mucosal blood vessels and maintains intestinal homeostasis. Through polysaccharide degradation and interactions with other microorganisms, *Bacteroides* generates a diverse array of metabolic products, with short-chain fatty acids (SCFAs) being among the most significant [68].

Evidence suggests that exposure to MC-LR may alter the normal functions of the intestinal microbiota in aquatic animals [69]. In this study, KEGG level 3 pathways showed that MC-LR significantly inhibited multiple metabolism-related functional pathways, including energy metabolism, nucleotide metabolism, biotin metabolism, and butyrate metabolism. This indicates that MC-LR may inhibit the corresponding metabolic functions of the intestinal microbiota. Meanwhile, it was observed that in the treatment group, the predicted level of the pathways related to lipopolysaccharide biosynthesis proteins in the gut microbiota was higher. Lipopolysaccharide is an endotoxin. If the functional pathway of lipopolysaccharide biosynthesis proteins is over-upregulated, bacteria will produce a large amount of lipopolysaccharide, which may lead to over-activation of the host immune system and uncontrolled inflammatory responses [70].

### 4.4. Effect of MC-LR on the Metabolism of Intestinal SCFAs

SCFAs are mainly produced by the fermentation of indigestible dietary fibers by bacteria of Firmicutes and Bacteroidetes. They not only improve intestinal health [71] and provide energy [72] but also serve as substrates or signaling molecules to regulate metabolism [73]. In this study, following MC-LR treatment, the concentration of SCFAs in the feces of GIFT tilapia was significantly reduced compared to the control group. This suggests that MC-LR may interfere with the normal metabolic process by inhibiting SCFA-producing bacteria, particularly those belonging to the Firmicutes and Bacteroidetes. Our findings indicate that exposure to MC-LR disrupts the intestinal microbiota structure of GIFT tilapia, which in turn affects its metabolic processes and compromises intestinal health.

### 4.5. Combined Analysis of Intestinal Flora, Biochemical Indexes, and SCFAs

Correlation analysis further elucidated the intricate relationships between the gut microbiota, biochemical indicators, and SCFA metabolism. In this study, *Acinetobacter* and *Bacteroides* exhibited significant negative correlations with multiple biochemical indicators, whereas *Aurantimicrobium* and *Pseudomonas* showed positive correlations. *Bacteroides* plays a crucial role in regulating fish metabolism, particularly in carbohydrate and lipid metabolism. Exposure to MC-LR can disrupt its normal function, leading to metabolic disturbances. Although *Escherichia-Shigella* is a pathogenic bacterium, a decrease in its abundance may impact the intestinal immune balance. Under stress conditions, *Pseudomonas* may mitigate oxidative damage caused by MC-LR through the regulation of metabolic pathways, production of antioxidant substances, or modulation of related gene expression. Furthermore, *Acinetobacter*, *Aquabacterium*, *Methyloversatilis*, and *Rhodococcus* were positively correlated with several SCFAs, indicating that they may possess unique metabolic pathways that facilitate SCFA generation or the conversion of environmental nutrients into SCFAs. Based on the correlation analysis results, we hypothesize that MC-LR may alter the composition of the intestinal microbiota via unknown mechanisms, leading to intestinal damage and impairing the normal physiological functions and metabolic activities of GIFT tilapia.

## 5. Conclusions

The results of this study indicate that intraperitoneal injection of MC-LR in GIFT tilapia induces oxidative stress, impairs immune function, alters the composition of the intestinal microbiota, and leads to intestinal damage and metabolic dysfunction. Additionally, we speculate that there is a correlation between the intestinal microbiota, SCFAs, and lipid metabolism in GIFT tilapia; however, the specific underlying mechanisms require further investigation. This represents one of the key directions for our future research. This study provides a theoretical foundation for a deeper understanding of the toxicological mechanisms of MC-LR.

## Data Availability

All data are available on request from the corresponding author.

## Supplementary Materials

The following supporting information can be downloaded at: https://www.mdpi.com/article/10.3390/antiox14030296/s1, **Figure S1:** (A) Venn Diagram. (B) rarefaction Curve; **Figure S2:** Measures of α and β diversity of the intestinal microbiota. (A) Shannon index. (B) Simpson’s index. (C) Chao1 index. (D) Pielou’s index. (E) The principal coordinate analysis of the two sample groups; **Figure S3:** KEGG level 2 pathways; **Figure S4:** KEGG level 3 pathways; **Table S1:** Gradient elution.
