# Supplementary material for: Study on the Toxicological Impacts of Intraperitoneal Microcystin-LR Injection on GIFT Tilapia (Oreochromis niloticus) Through Multi-Omics Analysis
Source: Antioxidants (Basel). 2025 Feb 28;14(3):296. doi: 10.3390/antiox14030296 (PMC11939458; doi:10.3390/antiox14030296)
Supplement: Supplementary file 1 [file antioxidants-14-00296-s001.zip › antioxidants-3443321-supplementary.pdf]

Supplementary material

Table S1. Gradient elution

Time（min）	A(%)	B(%)	Rate（mL/min）
0.00	75.00	25.00	0.300
0.50	75.00	25.00	0.300
13.00	45.00	55.00	0.300
15.80	20.00	80.00	0.300
15.90	75.00	25.00	0.300

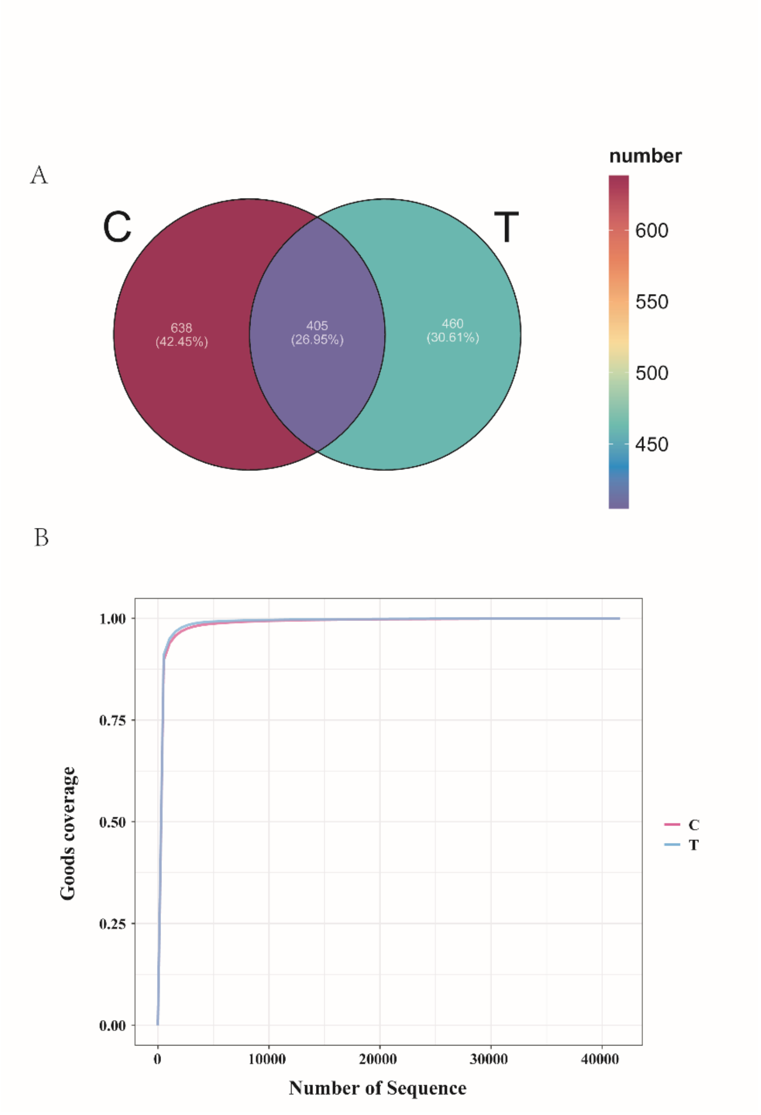

Figure S1. (A) Venn Diagram. (B) rarefaction Curve.

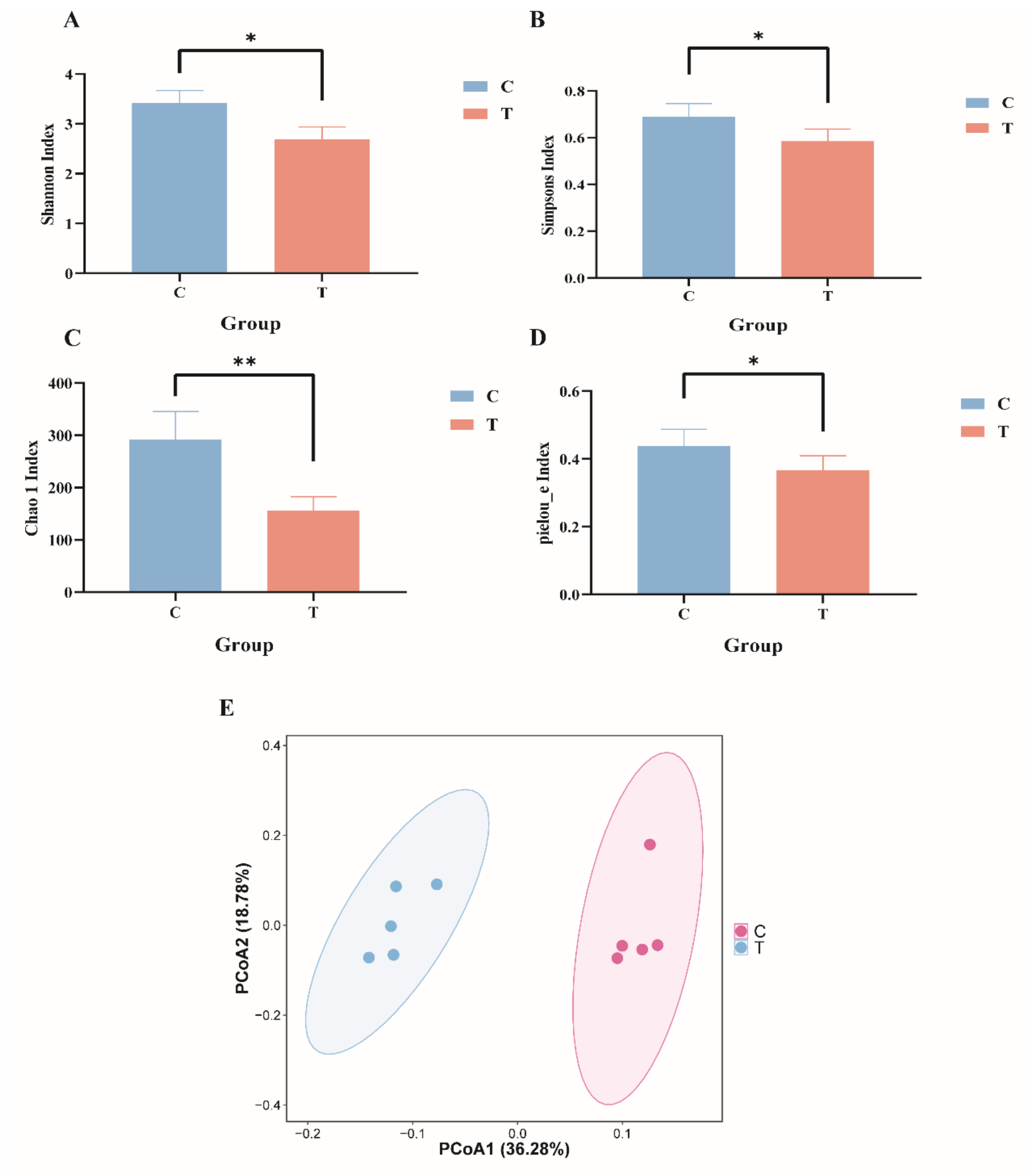

Figure S2. Measures of α and β diversity of the intestinal microbiota. (A) Shannon index. (B) Simpson's index. (C) Chao1 index. (D) Pielou's index. (E) The principal coordinate analysis of the two sample groups. *$P < 0.05$, **$P < 0.01$. Compared with the control group.

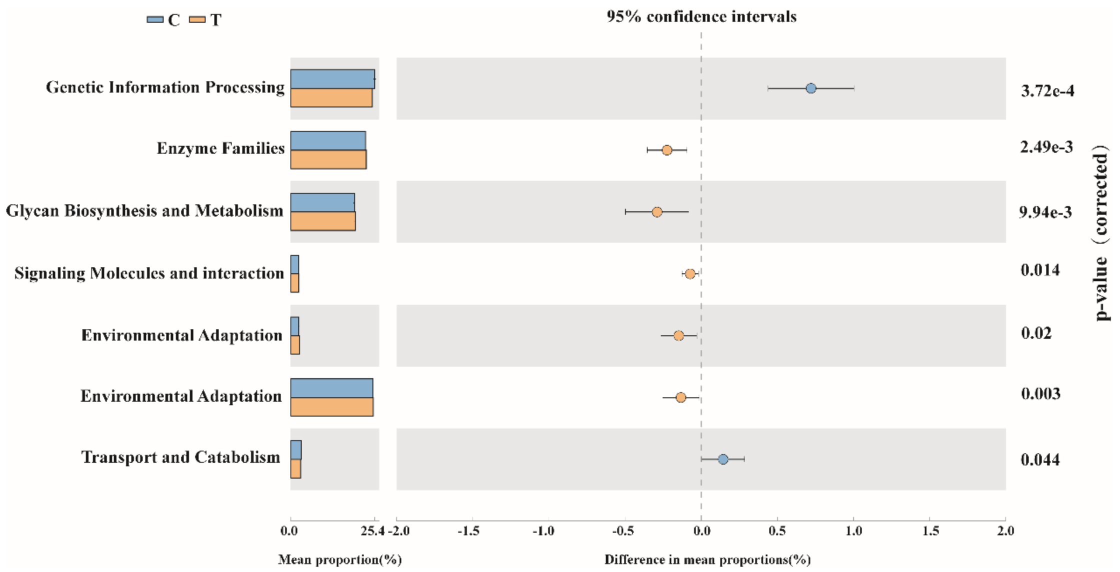

Figure S3. KEGG level 2 pathways.

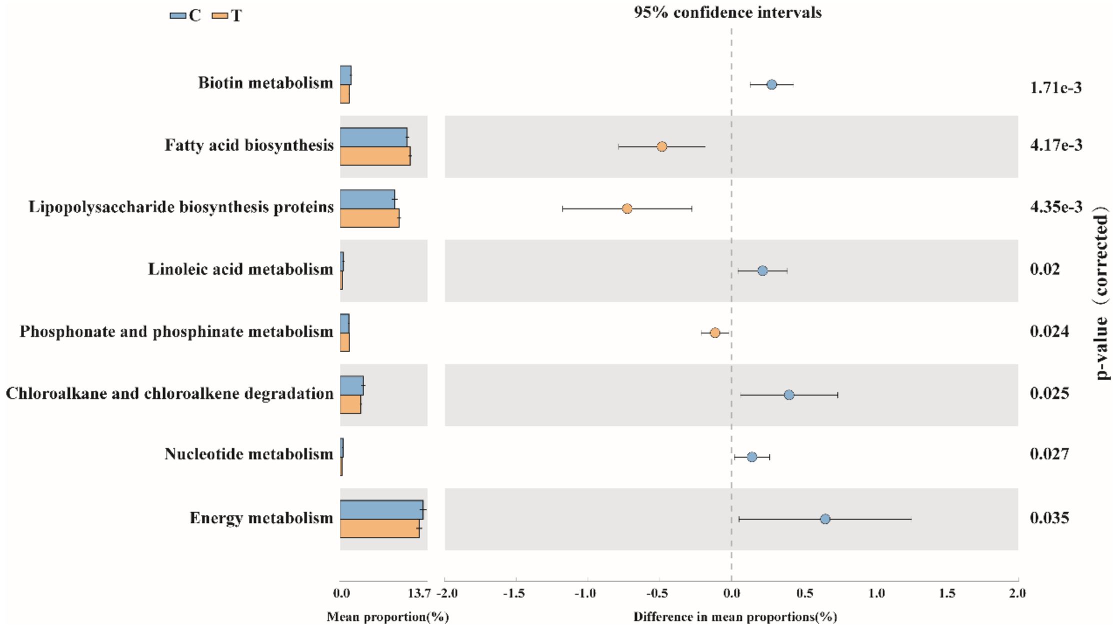

Figure S4. KEGG level 3 pathways.
